# Risk Factors for Stillbirth among Pregnant Women Infected with Syphilis in the Zhejiang Province of China, 2010–2016

**DOI:** 10.1155/2021/8877962

**Published:** 2021-02-02

**Authors:** Cui-Cui Duan, Xiao-Hui Zhang, Shan-Shan Li, Wei Wu, Li-Qian Qiu, Jian Xu

**Affiliations:** ^1^Women's Hospital of Zhejiang University School of Medicine, Hangzhou, China; ^2^The First Affiliated Hospital of Zhejiang University School of Medicine, Hangzhou, China; ^3^The Fourth Affiliated Hospital of Zhejiang University School of Medicine, Hangzhou, China

## Abstract

**Background:**

The World Health Organization estimated that about 1.36 million pregnant women suffered from syphilis in 2008, and nearly 66% of adverse effects occurred in those who were not tested or treated. Syphilis infection is one of the most common maternal factors associated with stillbirth.

**Objective:**

This study aimed to determine the risk factors for stillbirth among pregnant women infected with syphilis.

**Methods:**

In this retrospective study, data on stillbirth and gestational syphilis from 2010 to 2016 were extracted from the prevention of mother-to-child transmission (PMTCT) program database in the Zhejiang province. A total of 8,724 pregnant women infected with syphilis were included. Multiple logistic regression analysis was performed to determine the degree of association between gestational syphilis and stillbirth.

**Results:**

We found that the stillbirth percentage among pregnant women infected with syphilis was 1.7% (152/8,724). Compared with live births, stillbirth was significantly associated with lower maternal age, not being married, lower gravidity, the history of syphilis, nonlatent syphilis stage, higher maternal serum titer for syphilis, inadequate treatment for syphilis, and later first antenatal care visit. In multiple logistic analysis, nonlatent syphilis (adjusted odds ratio (AOR) = 2.03; 95% CI = 1.17, 3.53) and maternal titers over 1 : 4 (AOR = 1.78; 95% CI = 1.25, 2.53) were risk factors for stillbirth, and adequate treatment was the only protective factor for stillbirth (AOR = 0.16; 95% CI = 0.10, 0.25).

**Conclusions:**

Nonlatent syphilis and maternal titers over 1 : 4 were risk factors for stillbirth, and adequate treatment was the only protective factor for stillbirth.

## 1. Introduction

Stillbirth is a serious medical issue during childbirth. While, the overall stillbirths worldwide have decreased markedly, from 4 million in 1990 to 2.1 million in 2015 [1], stillbirth estimates vary widely across geographic areas. For example, the rates of stillbirth ranged from 1.2 per 1000 births in Iceland and 3.4 per 1000 births in Thailand to 27.6 per 1000 births in Pakistan and in South and Southeast Asia and 56.3 per 1000 births in South Sudan [1]. Studies have shown that women who have experienced the loss of a baby through stillbirth were more likely to suffer from psychological distress and a higher risk of recurrence of stillbirth compared to women without a history of fetal loss [2–5]. Thus, stillbirths place a huge burden on both the family and society.

Syphilis remains the most common congenital infection worldwide with tremendous consequences for both the mother and her developing fetus if it is left untreated. This infection can occur at any stage of pregnancy and during any trimester [6]. The economic and reproductive costs are enormous, as 25% of pregnancies may result in stillbirth, miscarriage, preterm birth, or other adverse pregnancy outcomes [7, 8]. In 2012, an estimated 930,000 syphilis infections in pregnant women caused 143,000 early fetal deaths and stillbirths around the world [9]. The wide adoption of syphilis screening and effective treatment with penicillin has made gestational syphilis entirely preventable [10–12]. However, sufficient intervention to prevent and treat gestational syphilis does not take place in many cases, especially in low-income and middle-income countries [13, 14].

In China, both stillbirth and syphilis place a large burden on maternal and infant health. The latest data showed that the average stillbirth rate in China was 8.8 per 1000 births from 2012 to 2014, and China ranked in the top five countries of the world in 2015 in the total number of stillbirths [15, 16]. Also, syphilis affects a large number of pregnant women in China. In 2013, 15,884 pregnant women diagnosed with syphilis delivered children in China, of which 14% suffered from serious adverse pregnancy outcomes including preterm delivery/low birth weight, congenital syphilis in newborns, and stillbirth/neonatal death [8, 17]. In 2011, the Chinese government initiated a national program for the prevention of mother-to-child transmission program, which continues until now. In this program, all pregnant women are recommended to receive syphilis screening, and appropriate treatment is provided to all positive women. The purpose of this study was to determine the risk factors for stillbirth among women infected with syphilis during pregnancy in the Zhejiang province. This province, which is located on the east coast of China, experienced rapid economic growth which attracted large migrant populations, and the prevalence of syphilis among pregnant women was 0.3%, according to a latest report [12]. Our aim was to gather additional epidemiological data for healthcare efforts focused on preventing stillbirth events in these mothers.

## 2. Methods

In this retrospective study, all pregnant women who were infected with syphilis and delivered babies from 2010 to 2016 were included, regardless of their birth outcomes. Stillbirth was defined as fetal death occurring at 28 weeks of gestation or more or death at delivery. Cases where the birthweight was equal or greater than 1000 g were included when the information on gestational weeks was missing. A questionnaire was used to collect all the information, including maternal sociodemographic characteristics, utilization of PMTCT and antenatal care services, pregnancy outcomes, and other factors such as previous pregnancy and birth history and marital status. We also extracted data from medical registries in hospitals and records in the antenatal care systems. Multiple births were excluded because they involve a higher risk of adverse pregnancy outcomes. All data were collected via the network reporting system. The medical staff in hospitals were responsible for data collection. Quality control was performed by researchers from a women's hospital at Zhejiang University that serves as a center for the PMTCT program. The study was approved by the ethics committee of this women's hospital (number: 2016–0005). All patient information was kept confidential in Women's Hospital, School of Medicine, Zhejiang University. Ethical approval was obtained from the Women's Hospital, School of Medicine, Zhejiang University (2016–0005).

### 2.1. Syphilis PMTCT

In the Zhejiang province, syphilis testing and counseling was routinely offered to all pregnant women at their first antenatal care visit during the period in which data have been collected. Positive results in both the toluidine red unheated serum test (TRUST)/syphilis rapid plasma reagin (RPR) and the *Treponema pallidum* particle agglutination (TPPA)/*pallidum* particle agglutination (TPHA) tests were used as diagnostic criteria for women with gestational syphilis. Women at high risk for syphilis were recommended to undergo screening more frequently. Before delivery, all women positive for syphilis were offered maternal serum testing again. Women with two courses of penicillin injections and at least two weeks off between the two courses were categorized into the adequate treatment group (2.4 million units of benzathine penicillin weekly injections for three weeks or 0.8 million units of procaine penicillin daily injections for 15 days per treatment course). Erythromycin and ceftriaxone replacement therapy was recommended for women who were allergic to penicillin. The follow-up and healthcare services for mothers and their infants were performed by the local women and children's hospitals.

### 2.2. Statistical Analysis

Data were analyzed using SPSS software v.16.0 for Windows. Categorical variables were presented as numbers and frequencies. The demographic characteristics of the women infected with syphilis were noted. The chi-square test was used to compare the prevalence of specific characteristics in women who did or did not suffer from stillbirths. The odds ratios (OR) and their 95% confidence intervals (CI) of the factors associated with stillbirth were estimated using univariate and multiple logistic regression models. *P* values under 0.05 were considered statistically significant. All tests were two-tailed. To count all births, women with one or more pregnancies during the study period were counted per pregnancy. Only women who were treated with two courses of penicillin injections were considered adequately treated. Maternal demographic characteristics, syphilis conditions, utilization of PMTCT, and antenatal care services were considered as factors that can affect stillbirth.

## 3. Results

### 3.1. General Characteristics

During the study period, 8,724 women were diagnosed with syphilis at some point in their pregnancy. The stillbirth percentage among pregnant women infected with syphilis was 17.4% (152/8,724). Some stillborn babies did not undergo congenital syphilis screening. The percentage of maternal age, marital status, gravidity, previous syphilis, syphilis stage, maternal serum titer for syphilis testing, adequate treatment, and the time of the first antenatal care visit differed significantly between women who had stillbirths and women who had live births (Table 1). Compared with live births, stillbirths in women were significantly associated in univariate analysis with lower maternal age, not being married, lower gravidity, the history of syphilis, nonlatent syphilis stage, higher maternal serum titer for syphilis, inadequate treatment for syphilis, and later first antenatal care visit (data not shown).

### 3.2. Factors Associated with Stillbirth

Using a multiple logistic regression analysis, we found that primary or secondary syphilis (adjusted OR (AOR) = 2.03; 95% CI = 1.17, 3.53) and maternal titers over 1 : 4 (AOR = 1.78; 95% CI = 1.25, 2.53) were significant risk factors for stillbirth, after controlling for the influence of maternal age, marriage, gravidity, previous syphilis, syphilis treatment, and first ANC (antenatal care visit, ANC). In addition, adequate treatment was the only protective factor for stillbirth (AOR = 0.16; 95% CI = 0.10, 0.25) (Table 2).

## 4. Discussion

In order to achieve the World Health Organization's goal of eliminating HIV infection and congenital syphilis in children by 2015, China faces many national challenges. In the second half of 2010, the Chinese government incorporated the national PMTCT plan for HIV, syphilis, and hepatitis B into the existing maternal and child healthcare system. In this study, data on stillbirth and gestational syphilis from 2010 to 2016 were extracted from this database. The rate of stillbirth among pregnant women infected with syphilis was 17.4 per 1000 births. Our estimate was not only slightly higher than the global average level (14.9 per 1000 live births) in 2015 but also double than China's national average (8.8 per 1000 births) from 2012 to 2014 [1, 15, 18]. Our national data were obtained from 441 hospitals in 326 urban districts and rural counties [15]. One of the probable reasons for the high rate of syphilis in the Zhejiang province may be attributed to the relatively complete infectious disease reporting system, where the doctor must enter syphilis cases into the system in time. In addition, the relatively developed economy of the Zhejiang province has attracted a large number of migrant workers who have relatively open view of sex, high mobility, weak awareness, and poor economic conditions, which increase the difficulty of clinical management. Also, the regional disparities in socioeconomic background, inclusion criteria, and healthcare services might lead to the differences in the prevalence of stillbirth in women with syphilis in different studies.

Stillbirth is closely related to syphilis particularly in low-income and middle-income countries, one of the reasons for it being the low detection rate of syphilis during pregnancy [19, 20]. Strengthening the focus on screening for syphilis has led to an 80% reduction in stillbirths, which is better than strategies that have been used to prevent malaria, HIV, and bacterial vaginosis infections in pregnant women in developing countries [21]. Previous studies have also showed that the odds ratio of stillbirth among pregnant women infected with syphilis ranges from 1.88 to 6.87, which is higher than that among pregnant women without syphilis infection, based on data from both developed and developing countries [22, 23]. Thus, based on these findings, we suggested to give a parity for syphilis screening for pregnant women.

In our study, higher maternal serum titers, primary or secondary syphilis stage, and lack of adequate therapy during pregnancy were independent risk factors for stillbirth. These results were consistent with other reports looking at stillbirths in pregnant women with syphilis in many countries including the United States [12, 17, 19, 21–25]. In most studies, high maternal titers, defined as serum titers over 1 : 8, were regarded as a risk factor for adverse pregnancy outcomes [19, 26]; however, we used a maternal titer threshold of 1 : 4 to define high titer, and it still correlated with greatly increased occurrence of stillbirth in these women. In the Zhejiang province, maternal titers were checked monthly to keep them under 1 : 4 or to achieve a 4-fold reduction in them by delivery. If not, these women were considered as having suffered from either treatment failure or reinfection and were suggested to undergo treatment again. The decline in titer varied with the severity of the disease and treatment the woman underwent. As previously reported, titers were usually higher and declined rapidly after treatment in primary and secondary syphilis [10, 27]. Accordingly, it was observed that a large percentage of women who suffered from stillbirth had primary and secondary syphilis.

The pregnancy outcomes could be greatly improved with adequate treatment. A large-scale meta-analysis indicated that the percentage of stillbirths and fetal loss is 26% for untreated maternal syphilis and 21% for maternal syphilis that was treated by the third trimester, whereas it dropped to 4% for nonsyphilitic mothers [24]. Unfortunately, in this study, not all infected women received adequate therapy. The percentage of adequate treatment was only 62% in women who gave birth to live children. In contrast, the percentage of adequate treatment was only 17% in women who had stillbirths. This phenomenon reflects the current barriers to gestational syphilis prevention or antenatal care in the Zhejiang province. Most women with syphilis were with low education, unemployed, had poor health awareness, or nonresidents, which exerted negative influences on their utilization of antenatal healthcare and syphilis intervention. At the national level, only 79% of infected women received antenatal care at or before 37 gestational weeks, and 55% of women who experienced stillbirth received no or late care [16]. In a study from India, out of the total women who had stillbirths, 56% had only one antenatal care visit with 6% of these women undergoing syphilis screening [28].

Screening and diagnosis are the first steps to prevent the transmission of syphilis from pregnant women to the developing fetus [29, 30]. Dual point-of-care tests available in the Zhejiang province are cost-effective. When seropositive pregnant women are identified, gestational care with syphilis therapy is offered as a protective factor for combating adverse pregnancy outcomes. In the Zhejiang province, 90 percentage of the women infected with syphilis are poorly educated and more than 40 percentage are unemployed. These might limit their adoption of PMTCT and antenatal care services for their poor awareness, which has been also observed in other areas [12]. In the United States, syphilis screening has even been advocated among asymptomatic, nonpregnant adults, and adolescents [31]. Thus, it is necessary to promote syphilis screening among all reproductive-age women.

This study has three main limitations that need to be taken into account. First, only pregnant women infected with syphilis and the ones who delivered babies between 2010 and 2016 were eligible for inclusion. Control group information of women that tested negative for gestational syphilis was not collected. Hence, we could not compare the incidence of stillbirth in women infected with syphilis and women without syphilis infection. Second, data for causes of stillbirth, which could be fetal, placental, external, or undetermined, were not collected. Many potential risk factors associated with stillbirths are modifiable and usually coexist, such as maternal demographic factors, maternal age, environment, nutrition, noncommunicable diseases, antenatal care, obstetric complications, and birth information. These issues were not discussed in our study and should be addressed and followed by adequate interventions. Finally, some stillbirth babies did not undergo syphilis screening. Even with these limitations, this study is important, as it was a large-scale multicenter study in a high-prevalence area. As stillbirth attributed to maternal syphilis has been poorly tracked and investigated in China, our results are valuable for further prevention efforts.

## 5. Conclusions

Maternal syphilis is strongly associated with adverse pregnancy outcomes. Mother-to-child transmission of syphilis is a public health issue due to high incidence of gestational syphilis and the negative influence it has on pregnancy outcomes. Early identification of syphilis is needed to improve pregnancy outcomes. Nonlatent syphilis and maternal titers over 1 : 4 were risk factors for stillbirth, and adequate treatment was the only protective factor for stillbirth.

## Figures and Tables

**Table 1:** Comparison of maternal sociodemographic characteristics and syphilis condition between women who had stillbirths and women who had live births.

Variables	Category	Stillbirth (*n* = 152)		Without stillbirth (*n* = 8,572)		*χ* ^2^	*P*
		*n*	%	*N*	%		
Maternal age (years)	<20	17	11.18	294	3.43	36.94	**<0.001**
	20–24	46	30.26	1959	22.85		
	25–29	45	29.61	3047	35.55		
	30–34	19	12.50	1925	22.46		
	≥35	25	16.45	1347	15.71		

Employment	Farmer	49	32.24	2891	33.73	1.28	0.735
	Business	12	7.89	598	6.98		
	Unemployed	63	41.45	3753	43.78		
	Unknown	28	18.42	1330	15.52		

Education	Primary	29	19.08	1288	15.03	5.65	0.130
	Middle	116	76.32	6461	75.37		
	College	6	3.95	745	8.69		
	Unclear	1	0.66	78	0.91		

Married		117	76.97	7845	91.52	39.64	**<0.001**
Unmarried		35	23.03	727	8.48		

Gravidity	1	43	28.29	1594	18.60	9.21	**0.002**
	≥2	109	71.71	6978	81.40		

Parity	0	70	46.05	3827	44.65	0.12	0.729
	≥1	82	53.95	4745	55.35		

Minority		10	6.58	541	6.31	0.02	0.893

Previous syphilis		25	16.45	3264	38.08	29.75	**<0.001**

Previous fetal loss		11	7.24	822	9.59	0.96	0.328
Syphilis stage	Latent	118	77.63	7068	82.45	21.57	**<0.001**
	Primary	16	10.53	326	3.80		
	Secondary	3	1.97	67	0.78		
	Tertiary	0	0	17	0.20		
	Unclear	15	9.87	1094	12.76		

Titer for TRUST/RPR >1 : 4 before delivery		59	38.82	1820	21.23	27.33	**<0.001**

Treated		76	50.00	7296	85.11	140.62	**<0.001**

Adequately treated		26	17.11	5310	61.95	126.423	**<0.001**

First antenatal care visit	≤12	42	27.63	4087	47.68	69.92	**<0.001**
	13–27	47	30.92	3082	35.95		
	≥28	51	33.55	1111	12.96		
	Unclear	12	7.89	292	3.41		

*Note.* Missing information was not included. Minority: in China, the minority refers to ethnic groups other than the Han ethnic group. The bold values indicate statistical significance.

**Table 2:** Multiple logistic regression analysis on associations between gestational syphilis and stillbirth.

Variable		Crude OR (95% CI)	Adjusted OR (95% CI)	*P* value
Syphilis stage	Latent	Ref	Ref	**0.043**
	Primary or secondary	2.94 (1.72, 5.01)	2.03 (1.17, 3.53)	
Maternal titer >1 : 4		2.35 (1.69, 3.28)	1.78 (1.25, 2.53)	**0.001**
Completely treated		0.13 (0.08, 0.19)	0.16 (0.10, 0.25)	**<0.001**

*Note.* Missing information was not included; OR, odds ratio; CI, confidence interval. The bold values indicate statistical significance.

## Data Availability

The datasets analyzed during the current study are not publicly available and are available from the corresponding author upon request.
